# Quantitative Magnetic Resonance Imaging of the Forearm in Myotonic Dystrophy Type 1

**DOI:** 10.3390/tomography11120136

**Published:** 2025-12-05

**Authors:** Sydney Eierle, Tanja Taivassalo, Hyunjun Park, Korey D. Cooke, Zahra Moslemi, Sean C. Forbes, Glenn A. Walter, Krista Vandenborne, S. H. Subramony, Donovan J. Lott

**Affiliations:** 1 Department of Applied Physiology and Kinesiology, University of Florida, Gainesville, FL 32610, USA; scveierle@gmail.com; 2 Department of Physiology & Aging, University of Florida, Gainesville, FL 32610, USA; ttaivassalo@ufl.edu (T.T.);; 3 Department of Physical Therapy, University of Florida, Gainesville, FL 32610, USA; hyunjun.paul.park@gmail.com (H.P.); scforbes@phhp.ufl.edu (S.C.F.);; 4 Kinesiology and Health Studies, University of Regina, Regina, SK S4S 0A2, Canada; 5 Department of Neurology, University of Florida, Gainesville, FL 32610, USA

**Keywords:** quantitative magnetic resonance imaging, fat fraction, transverse relaxation time, upper limb, myotonic dystrophy type 1, myotonia

## Abstract

Introduction: Myotonic dystrophy type 1 is the most prevalent muscular dystrophy in adults, characterized by weakness, impaired functional abilities, and myotonia. However, little is known about the relationship between quantitative MRI measures (fat fraction and T_2_ relaxation time) and clinical findings of the upper extremity. This study assessed forearm muscle structure in patients with myotonic dystrophy using quantitative MRI and correlated these measures with strength, function, and handgrip myotonia. Materials and Methods: Eighteen adults with myotonic dystrophy type 1 underwent MRI using three-point Dixon and T_2_ spin echo imaging of the forearm. Results: The average fat fraction and T_2_ relaxation time were greatest in the flexor digitorum profundus (26.7% and 55.6 ms, respectively). Correlations were found between quantitative MRI values and clinical tests of strength (r = −0.61 to −0.92, *p* < 0.01), function (r = −0.64 to −0.83, *p* < 0.01), and handgrip myotonia (r = 0.48, *p* < 0.05). Overall, the anterior forearm fat fraction values showed higher correlations with strength and function compared to those of the posterior forearm. Discussion: Our results support the use of quantitative MRI measures to assess forearm disease pathology and show potential to monitor the effectiveness of therapeutic treatments in patients with myotonic dystrophy type 1.

## Simple Summary

Myotonic dystrophy type 1 causes weakness, impaired movement, and myotonia. Little is known about MRI measures that quantify muscle fat infiltration and inflammation of the arm in these patients. We examined how these MRI measures in the forearm were related to strength, functional ability, and myotonia. Muscle fat and inflammation were greatest in specific muscles, and the anterior forearm had more muscle fat infiltration than the posterior forearm. The MRI measures correlated with measures of strength, function, and myotonia. These quantitative MRI measures in the forearm show potential to monitor the effectiveness of treatments in these patients.

## 1. Introduction

Myotonic dystrophy type 1 (DM1) is a highly variable multi-systemic disorder caused by an expansion of a CTG trinucleotide repeat in the noncoding region of the *DMPK* gene [1]. DM1 is the most common form of muscular dystrophy in adults, with an estimated prevalence of 1:8000 [2]. Patients with DM1 have shown variable age at onset, clinical phenotypes and disease severity, which are at least partly related to the size of the CTG-repeat expansion [3,4]. The clinical characteristics of the disease include myotonia (sustained muscle contraction), progressive muscle weakness, cataracts, endocrine system disturbances, arrhythmia, and central nervous system impairments [1].

Distal muscle weakness of the upper and lower extremities is a core feature of DM1 [2,5,6,7,8]. In particular, the forearm musculature is significantly impacted by both weakness and handgrip myotonia. These impairments often impede the performance of activities of daily living (ADLs) such as using utensils, grasping doorknobs, putting on clothes, and utilizing other household equipment [1,9,10]. Patients with DM1 have reported that the most prevalent difficulties associated with the disorder were those of the hands and arms, followed by fatigue and myotonia [11]. Therefore, it is important to evaluate the muscle structure of the forearm to understand upper limb impairments and the impact of potential therapeutic interventions on these features.

To determine the effectiveness of therapeutic interventions for patients with DM1, it is essential to have outcome measures and biomarkers that are sensitive to treatment effects and objectively quantifiable. Clinical assessments of strength and function are dependent on a patient’s effort and motivation and lack sensitivity to small pathophysiological changes in muscle [12]. Quantitative magnetic resonance imaging (qMRI) is more sensitive and reliable than clinical measures, as seen in studies that have quantified disease severity and progression in the upper and lower extremities of patients with Duchenne muscular dystrophy [13,14]. More limited experience with qMRI in DM1 has shown that relative to unaffected individuals, patients with DM1 demonstrate higher values for muscle fat, inflammation, and edema, measured by fat fraction (FF) and T_2_ relaxation time [7,8,15]. Heskamp et al. used qMRI to assess the longitudinal changes in FF and T_2_ relaxation time throughout a behavioral intervention targeting physical activity in patients with DM1 [16]. These studies, however, focus primarily on disease severity and progression in the lower extremities rather than the upper extremities. While imaging studies of the lower extremities in those with DM1 are more common likely due to their role in general mobility, qMRI studies of the upper limb muscles are urgently needed to monitor therapeutic interventions and provide clinically meaningful endpoints in DM1, given the impact of upper limb deficits in this disease. To our knowledge, only two previous studies have evaluated MRI findings of the upper limbs in patients with DM1 [5,6]. However, these clinical studies used ordinal scales or semi-quantitative MRI methods to evaluate skeletal muscle pathology and measure overall disease severity. Further investigation using qMRI is necessary to analyze functionally important upper extremity muscles, such as those of the forearm.

The objective of this study was to assess forearm muscle pathology in patients with DM1 using qMRI and determine how these qMRI measures (FF and T_2_ relaxation time) correlate with measures of function, strength, and handgrip myotonia. Based on the aforementioned studies, we hypothesized that FF and T_2_ relaxation time would negatively correlate with forearm muscle strength and function assessments and positively correlate with the severity of handgrip myotonia [5,6]. We also believed that FF and T_2_ values would be greater in the anterior forearm compared to the posterior forearm. Lastly, we hypothesized that forearm muscle pathology assessed by both FF and T_2_ relaxation time would positively correlate with the size of CTG repeat expansion as has been reported with semiquantitative MRI [5].

## 2. Materials and Methods

Individuals with a confirmed diagnosis of DM1 within the age range of 18–55 years were invited to participate in the study through clinic visits and advertisements. We collected clinical data including age, sex, age at diagnosis, age at symptom onset, body mass index (BMI), and number of CTG repeats (Table 1). To determine CTG-repeat length, a blood sample of approximately 8 mL was taken from each patient and analyzed by Athena Diagnostics (Marlborough, MA, USA). Direct testing for the repeat expansion mutation was performed by PCR amplification of the repeat region followed by high-resolution electrophoresis to determine the number of repeats. Southern blot analysis was used, as necessary, to confirm the homozygosity of normal alleles and to detect the number of repeats in expanded alleles.

Exclusion criteria included: (i) other neurologic or psychiatric disorders; (ii) coexistence of other neuromuscular disorders; (iii) untreated thyroid dysfunction; (iv) liver or kidney disease; (v) treatments with supplemental anabolic hormones, such as testosterone; (vi) treatment with an anti-myotonia medication (i.e., mexiletine) within the previous 8 weeks; and/or (vii) drug or alcohol abuse. We also excluded female participants who were pregnant or trying to become pregnant, and all patients with any contraindication to MRI (e.g., pacemakers, metal implants in the upper extremities, and claustrophobia).

The study protocol was approved by the Institutional Review Board of the University of Florida and written informed consent was obtained from all participants.

### 2.1. Clinical Assessments

All patients underwent clinical tests to assess muscle strength using quantitative muscle testing (QMT) [17,18,19] and degree of myotonia in the hand using video hand opening time (vHOT) [20,21]. Additionally, the Upper Extremity Functional Index (UEFI) questionnaire was given to each patient to evaluate functional abilities [22,23].

Measurements of strength by QMT included the long finger flexor (LFF) and handgrip (HG) tests using a handgrip dynamometer [17,19]. For the LFF and HG strength tests, each patient was seated with shoulder neutral, elbow in 90 degrees of flexion and wrist neutral. The handgrip Jamar was positioned at the second and last position for the HG and LFF tests, respectively. For both tests, the patient was asked to perform two separate maximal muscle contractions for 3–5 s with approximately a 10-s rest between each trial, and the strongest force generated was used for further analysis. Predicted strength for handgrip was also calculated as a percentage based on the maximal values from each subject’s handgrip relative to normative data [24].

Each patient completed the UEFI questionnaire to assess their upper limb functional ability [22,23]. The UEFI consists of 20 questions relating to physical or daily activities, each with a 5-point scale (0 = Extremely difficult or unable to perform activity to 4 = No difficulty performing the specific activity). The highest score possible is 80 points, indicating best functional status.

Myotonia of the hand was assessed by vHOT [20,21]. In brief after a 10-min rest, the patients placed their right hand on a flat surface and were asked to make a tight fist for 3 s and then open the hand as quickly as possible. Two trials were performed by each participant with each trial being video recorded. The time required for hand opening until all fingers and the thumb had actively extended as far as possible was determined by a single rater from the video recordings. The first trial was chosen to be used for scoring purposes unless it was deemed unacceptable due to the patient not following the instructions appropriately (i.e., opening the hand too early, moving the forearm and/or wrist excessively when opening the hand, etc.). If the first trial was not deemed acceptable, the second trial was then used for scoring purposes.

### 2.2. MRI Acquisition

All MRI measurements were performed using a 3.0-T whole body MRI scanner (Philips Achieva Quasar Dual 3T). The subjects laid in the scanner in the head-first supine position, with the right forearm in a neutral position. Padded supports were used to maintain a fixed position of the forearm and to ensure the patient was comfortable throughout the scans. An 8-channel flexible surface coil was placed optimally at the largest cross-sectional area of the forearm with the proximal field of view set at the radial head.

Multi-slice T_2_ spin echo axial images were used to assess muscle composition. T_2_ MRI is influenced by inflammation/edema and fatty tissue infiltration and has been used to detect subclinical changes in patients with muscular dystrophy [25,26]. Five equally spaced TEs from 20 to 100 ms, a TR of 3000 ms, and 7 mm slice thickness were used for 10 slices. In addition, multi-slice three-point Dixon images were acquired to quantify fat fraction. These images used a 2D multi-slice, single echo, gradient echo sequence run three times each with a different echo time (TE1 = 4.6 ms, TE2 = 5.6 ms, TE3 = 6.6 ms), a flip angle of 20 degrees, and 4 mm slice thickness for 25 slices. Images were acquired with a long repetition time (TR = 430 ms) to minimize the effects of T_1_-weighting. The validity of these sequences for FF determination has been previously confirmed in dystrophic muscle [27].

### 2.3. MRI Image Analysis

To produce T_2_ values, pixel-by-pixel T_2_ maps of the forearm muscles were created for each slice by using a monoexponential decay model [28] with custom-written software (Interactive Data Language, IDL). Regions of interest (ROI) were manually drawn on the T_2_ maps while staying ~1–2 mm inside the image border for five forearm muscles: flexor digitorum profundus (FDP), flexor digitorum superficialis (FDS), flexor pollicis longus (FPL), extensor pollicis brevis (EPB), and abductor pollicis longus (APL). For each muscle, the most proximal to most distal slices that had a clear representation and identifiable muscle boundaries were chosen for analysis (average number of slices for all five muscles = 5.5). Mean T_2_ values for each of the five forearm muscles were then calculated within each of the five ROIs, and the average across all the slices was determined for each muscle. Separate ROIs were drawn for the anterior and posterior forearm as well.

FF analyses included custom-written IDL software generating pixel-wise FF maps from the water and fat images. For the same five forearm muscles analyzed for the T_2_ measures, ROIs were drawn on the FF maps while staying 1–2 mm inside the image border to avoid including intermuscular tissue. For each muscle, the most proximal to most distal slices which had a clear representation and identifiable muscle boundaries were chosen for analyses (average number of slices for all five muscles = 12.1). The average FF was determined for all pixels within the ROI, and the average across all slices was used for each muscle’s FF.

### 2.4. Statistical Analysis

Statistical analyses were performed using IBM SPSS Statistics Version 30.0. Given the expected limited sample size from a patient population with a rare disease (*n* < 30), non-parametric Spearman rank correlation coefficients were determined for FF and T_2_ values of the forearm muscles with clinical strength and function results. Statistical significance was specified as *p* < 0.05. Data are presented as mean ± SD.

## 3. Results

### 3.1. Demographics and Clinical Assessments

We evaluated the right upper extremity of eighteen patients with DM1 (7 males and 11 females with one of the females being left-hand dominant). The mean age was 36.2 ± 12.3 years, and the mean age of symptom onset was 20.8 ± 9.5 years (Table 1). The mean number of CTG repeats was 516 ± 300.

Strength and function test results are shown in Table 2. The mean handgrip strength was 13.5 ± 7.4 kg, mean percent predicted handgrip strength was 36.5 ± 20.9%, and long finger flexor strength was 7.6 ± 4.3 kg. The average UEFI score was 57.9 ± 15.8. Additionally, all patients demonstrated the presence of handgrip myotonia by evidence of a delayed release of the handgrip with a mean hand opening time of 18.7 ± 13.0 s.

### 3.2. MRI Findings

**Figure 1:**
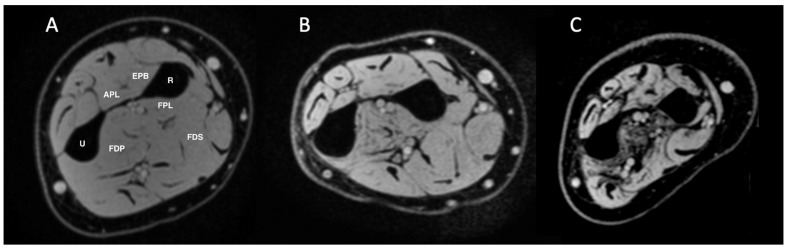
Representative three-point Dixon images from the forearm in a patient with mild disease presentation with little to no muscle pathology (**A**, Subject 11), in a patient with moderate disease presentation with muscle pathology noted in at a few areas and/or muscles (**B**, Subject 1), and in a patient with severe disease presentation with pathology noted to a large extent in more than one muscle (**C**, Subject 6). FDP = flexor digitorum profundus; FDS = flexor digitorum superficialis; FPL = flexor pollicis longus; EPB = extensor pollicis brevis; APL = abductor pollicis longus; U = ulna; R = radius.

**Figure 2:**
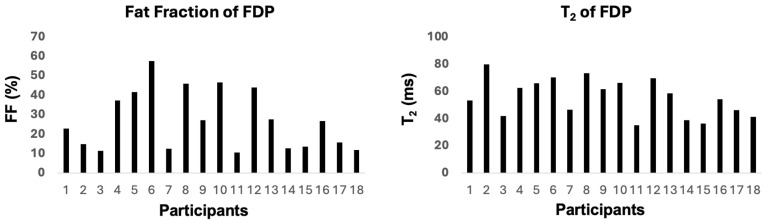
Fat fraction (FF) and T_2_ values from the flexor digitorum profundus (FDP) muscle for each of the 18 participants.

Quantitative MRI data were acquired from all 18 patients for five forearm muscles: FDP, FDS, FPL, EPB, and APL. Three-point Dixon images in a patient with mild disease, a patient with moderate disease, and a patient with severe disease are shown in Figure 1. The average FF and T_2_ values are displayed in Table 3 and Table 4, respectively. FF and T_2_ values showed a wide variability of muscle pathology between subjects with the greatest variability noted in the FDP as shown in Figure 2. Across all subjects, the average FF value was highest in the FDP (26.7%), followed by the FPL (22.1%) and APL (18.1%). Similarly, the average T_2_ relaxation time was noted to be longest in the FDP for all subjects except three (subjects 9, 11, and 16). For subjects 9 and 16, the FPL had the highest T_2_ value. For subject 11, the APL had the highest T_2_ value, followed by the FPL and then the FDP.

### 3.3. Relationship Between MRI and Clinical Findings

Forearm FF and T_2_ values presented strong correlations with measures of strength and function (Table 5 and Table 6). FDP FF values showed notably high correlations for all QMT tests (HG: r = −0.82; Predicted HG: r = −0.82; LFF: r = −0.79; all *p* < 0.01) and the UEFI (r = −0.77, *p* < 0.01). FDP T_2_ values also significantly correlated with these measures of strength and function (HG: r = −0.75, *p* < 0.01; Predicted HG: = −0.81, *p* < 0.01; LFF: r = −0.66, *p* < 0.01; UEFI: r = −0.55, *p* < 0.05). FPL FF showed a negative correlation with the Predicted HG (r = −0.48, *p* < 0.05), but it did not show a significant correlation with either LFF or HG tests. On the other hand, FPL T_2_ values showed strong negative correlations with HG and Predicted HG (r = −0.73, −0.82, respectively; both *p* < 0.01) and a moderate correlation with LFF (r = −0.66, *p* < 0.01).

As shown in Table 5, the anterior FA FF showed a stronger correlation with HG (r = −0.82, *p* < 0.01), Predicted HG (r = −0.77, *p* < 0.01), and LFF (r = −0.74, *p* < 0.01) strength compared to the posterior FA FF (HG: r = −0.60, *p* < 0.05; Predicted HG: r = −0.65, *p* < 0.01; LFF: r = −0.46, *p* = 0.073). However, similar correlations were found between anterior and posterior FA T_2_ values and clinical tests (Table 6). Additionally, the anterior FA FF showed a stronger correlation with UEFI (r = −0.81, *p* < 0.01) relative to that of the posterior FA (r = −0.63, *p* < 0.05).

We also found that the FDP T_2_ values positively correlated with the vHOT (r = 0.48, *p* < 0.05). Although there were no significant correlations between other qMRI values and the vHOT, there was a trend for correlations between FDP FF and the vHOT (*p* = 0.061) and between the anterior FA T_2_ and the vHOT (*p* = 0.064). Lastly, we did not find any significant correlations between qMRI findings and the length of CTG repeats or the age of diagnosis/noticed symptoms.

## 4. Discussion

This study demonstrated the utility of qMRI measures (FF and T_2_ relaxation time) to assess the muscle structure of the distal upper extremities in patients with DM1, shown by high FF and T_2_ values in the forearm of individuals with DM1 compared to unaffected individuals from other studies [29,30]. More importantly, these qMRI variables correlated with clinical measures of forearm strength, function, and handgrip myotonia, indicating the potential use of qMRI as an endpoint in DM1 studies.

qMRI has been used to evaluate pathological changes in several other muscle disorders [13,14,31,32,33,34,35]. Changes in FF may be used to assess therapeutic treatments that focus on improving muscle strength and function [34,35]. Similarly, increased T_2_ relaxation time, which is indicative of edema caused by inflammation, may be used as an early marker of pathologic changes in muscles of patients with neuromuscular disease [8,36,37]. Through the widespread usage of qMRI, it is evident that quantitative imaging is a promising objective assessment for disease progression and treatment response for individuals with DM1 [7,8,15].

### 4.1. Increased FF and T_2_ Values in DM1 Forearm

Similarly to the findings of Sugie et al. [5] and Hayashi et al. [6], we found the FDP and FPL muscles to be most affected in the forearm of people with DM1. As shown in Table 3 and Table 4, our muscular evaluations are based on quantified FF and T_2_ values, while the other two studies provide MRI findings classified according to an ordinal (Mercuri) [38] or semi-quantitative (Fischer) scale [39]. Other notably affected muscles were the FDS (patient 14), EPB (patient 11), and APL (patients 3 and 4), which may be associated with weakness in finger flexion and handgrip myotonia. Additionally, the anterior forearm had slightly higher average FF values compared to the posterior forearm, which could be due to the largely affected flexor muscles, such as the FDP, FPL, and FDS. Therefore, future clinical trials may want to focus on the impact that therapeutic interventions have on the anterior compartment to address the difficulties of fine motor control and the performance of ADLs in patients with DM1 [9,10,11].

### 4.2. Contribution of FF and T_2_ to Strength and Function Impairments

**Figure 3:**
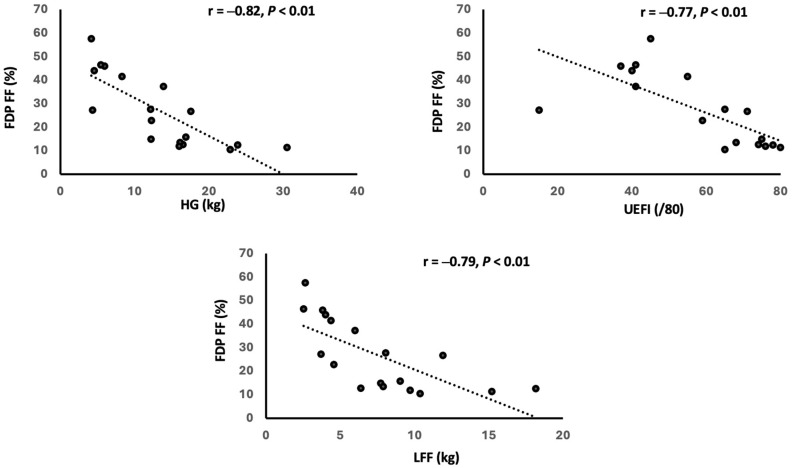
Correlation of MRI FF findings and clinical assessments. FDP = flexor digitorum profundus; FF = fat fraction; HG = handgrip strength; LFF = long finger flexor; UEFI = Upper Extremity Functional Index.

**Figure 4:**
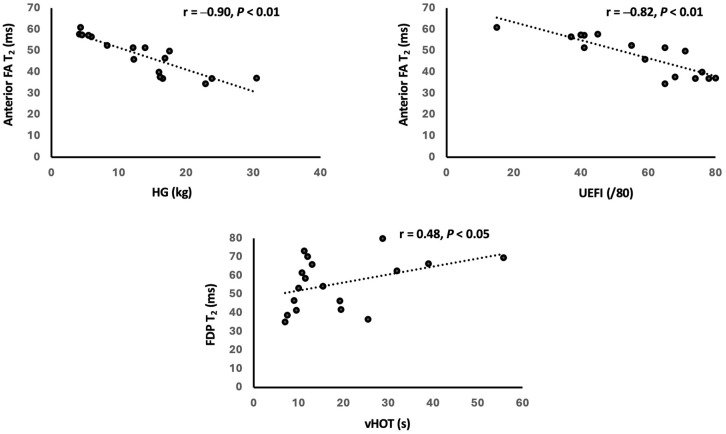
Correlation of MRI T_2_ findings and clinical assessments. FA = forearm; HG = handgrip strength; UEFI = Upper Extremity Functional Index; FDP = flexor digitorum profundus; vHOT = video hand opening time.

While Sugie et al. [5] and Hayashi et al. [6] have shown correlations between semi-quantitative MRI findings and forearm muscle weakness, this study demonstrated notably high correlations between qMRI FF and T_2_ values and forearm strength and function (Table 5 and Table 6; Figure 3 and Figure 4). Therefore, qMRI measures may be a valuable tool to quantitatively evaluate forearm strength and function in patients with DM1.

Building upon the findings of Sugie et al. [5] and Hayashi et al. [6] that focused on individual muscles, our study evaluated individual muscles as well as the anterior and posterior FA to determine DM1’s impact on different FA compartments. The anterior FA FF values showed prominent correlations with handgrip strength and long finger flexor strength as well as with the UEFI. These findings show that the anterior FA may be more affected in patients with DM1, signifying distal arm weakness and prominent involvement of the wrist and finger flexors [40,41]. Interestingly, the FDP was the only muscle to show a significant correlation between T_2_ relaxation time and handgrip myotonia, which was assessed using vHOT (Figure 4). While the contributing factors to myotonia extend beyond skeletal muscle pathology, further investigation is needed to address the effect that FDP has on handgrip myotonia. Using qMRI to quantify pathology in individual muscles and compartments may help in the development of specific treatment plans for the affected musculature such as exercises using silicone-based putty for resistance and endurance training [42,43].

### 4.3. Correlation of CTG Size with FF and T_2_ Values

Unlike Sugie et al. [5] but analogous to Hayashi et al. [6], we found no correlation between forearm FF and T_2_ values and CTG repeat length. This could be because our cohort of subjects had a narrower range of CTG-repeats compared to that of Sugie et al. The lowest from our sample was 111 (patient 15), and the highest was 1033 (patient 10). This range contrasts with that of Sugie et al. whose patients’ CTG-repeat lengths ranged from 100 to 2300, and several of these individuals had much higher repeat lengths than in our sample. Another possible reason is that the correlation found in the Sugie et al. study was between an overall MRI involvement score (analyzed from an ordinal scale) and CTG-repeat length, while our study focuses on the correlation between quantifiable FF and T_2_ values and CTG-repeat length. Therefore, further investigation is necessary to examine any relationship between qMRI measures and CTG-repeat length.

### 4.4. Study Limitations

Our study had some limitations. First, while no unaffected controls were included in this study, our findings can be compared with the results from other qMRI studies involving healthy controls. From these studies, it is clear that our patients demonstrated elevated FF and T_2_ values well above the healthy population [29,30]. A second limitation was the limited resolution of the imaging system that causes a partial volume effect. However, this effect was minimized by selecting specific ROIs on each forearm region. Third, the results from this single-center study only involving 18 adult participants with DM1 does limit generalizability. Lastly, our study was not a longitudinal study; therefore, we recommend additional studies to assess the sensitivity of qMRI measures over time when assessing the progression of DM1 in individual patients.

## 5. Conclusions

This is the first study to use qMRI to evaluate the distal upper extremity of patients with DM1. qMRI FF and T_2_ values show significant correlations with various clinical assessments of forearm strength, function, and handgrip myotonia. The anterior forearm appears to be a largely affected compartment in the upper extremity and should be further investigated. Our results suggest qMRI is a valuable method to evaluate the muscle structure and the efficacy of therapeutic interventions in patients with DM1.

## Figures and Tables

**Table 1:** Patient demographics.

Subject	Sex	Age (Years)	Age of Diagnosis (Years)	Age of Symptom Onset (Years)	BMI (kg/m^2^)	CTG Repeat
1	M	25.7	18.0	12.5	29.2	553
2	M	51.7	40.0	37.0	21.5	153
3	M	22.7	17.0	12.0	21.9	270
4	F	42.3	37.0	29.0	26.7	443
5	F	35.1	32.0	23.0	22.5	590
6	M	31.2	16.5	16.5	30.2	n.a.
7	F	26.2	19.0	18.0	20.5	393
8	M	42.9	37.0	20.0	29.5	n.a.
9	F	47.4	36.0	25.0	21.5	740
10	F	35.1	18.0	13.0	18.9	1033
11	F	21.1	19.0	15.5	22.7	513
12	M	48.2	32.0	29.0	24.5	134
13	F	55.9	25.0	40.0	26.7	973
14	F	18.7	10.0	12.5	18.1	440
15	F	54.4	n.a.	34.0	25.7	111
16	F	26.5	15.0	11.5	34.1	n.a.
17	M	42.5	11.0	9.0	18.6	953
18	F	23.3	22.0	17.0	31.8	440
Mean		36.2	23.8	20.8	24.7	515.9
SD		12.3	9.8	9.5	4.8	299.8

n.a.—Not available.

**Table 2:** Clinical assessment outcomes of patients with DM1.

Subject	HG (kg)	HG Pred (%)	LFF (kg)	UEFI (/80)	vHOT (s)
1	12.3	22.3	4.6	59	10.0
2	12.2	23.7	7.7	75	28.8
3	30.5	55.5	15.2	80	19.5
4	13.9	43.4	6.0	41	32.0
5	8.3	24.6	4.4	55	13.0
6	4.2	7.5	2.6	45	12.0
7	23.9	70.3	18.2	78	9.0
8	6.0	11.2	3.8	37	11.3
9	4.3	15.3	3.7	15	10.8
10	5.5	16.3	2.5	41	39.0
11	22.9	71.5	10.4	65	7.0
12	4.6	9.1	4.0	40	55.8
13	12.1	46.6	8.1	65	11.5
14	16.5	51.7	6.4	74	7.5
15	16.1	53.9	7.9	68	25.5
16	17.6	51.8	11.9	71	15.5
17	16.9	31.8	9.0	n.a.	19.3
18	16.0	50.0	9.7	76	9.5
Mean	13.5	36.5	7.6	57.9	18.7
SD	7.4	20.9	4.3	18.5	13.0

All values were taken from the right arm. Quantitative muscle testing (QMT) measures include: HG = handgrip strength; pred = predicted; LFF = long finger flexor; and pinch. UEFI = Upper Extremity Functional Index; vHOT = video hand opening time. n.a.—Not available.

**Table 3:** Fat fraction values of patients with DM1.

Muscle FF (%)	FDP	FDS	FPL	EPB	APL	Ant.	Post.
Subject							
1	22.8	13.1	20.6	11.3	12.1	19.4	21.0
2	14.9	10.7	12.2	9.7	8.7	15.4	13.5
3	11.4	10.1	12.4	12.7	14.5	12.8	14.8
4	37.3	18.0	21.8	37.7	43.0	n.a.	n.a.
5	41.6	12.5	44.1	23.2	16.9	31.9	23.1
6	57.6	16.1	18.2	19.3	18.8	34.6	32.4
7	12.5	n.a.	19.1	n.a.	n.a.	n.a.	n.a.
8	45.9	16.5	20.5	14.6	15.3	31.3	23.8
9	27.3	18.5	32.9	14.2	17.7	31.3	20.9
10	46.5	14.9	20.7	11.8	13.3	28.0	18.6
11	10.5	12.1	12.8	13.5	11.4	13.3	13.9
12	44.0	27.6	45.7	n.a.	43.9	31.2	32.1
13	27.7	17.1	19.8	15.0	18.1	25.6	20.6
14	12.7	14.0	13.8	n.a.	11.3	15.6	15.5
15	13.5	12.8	14.9	12.0	11.7	16.9	17.5
16	26.8	15.2	30.9	n.a.	21.0	25.4	26.0
17	15.7	14.7	21.8	16.6	14.3	21.0	20.3
18	11.8	13.2	15.9	15.1	15.5	16.2	17.4
Mean	26.7	15.1	22.1	16.2	18.1	23.1	20.7
SD	15.2	4.0	10.0	7.1	10.1	7.6	5.8

All values were taken from the right arm. FDP = flexor digitorum profundus; FDS = flexor digitorum superficialis; FPL = flexor pollicis longus; EPB = extensor pollicis brevis; APL = abductor pollicis longus; Ant. = anterior forearm; Post. = posterior forearm. n.a.—Not available.

**Table 4:** T_2_ values of patients with DM1.

Muscle T_2_ (ms)	FDP	FDS	FPL	EPB	APL	Ant.	Post.	Avg.
Subject								
1	53.3	44.6	44.4	42.5	44.1	45.9	41.2	43.6
2	79.8	69.4	54.9	42.1	38.5	n.a.	n.a.	n.a.
3	41.8	34.2	38.2	33.8	32.3	37.2	32.7	34.9
4	62.4	55.6	45.3	44.1	40.5	51.4	39.9	45.6
5	65.9	52.2	57.9	52.4	42.8	52.5	44.2	48.3
6	70.1	59.0	69.3	56.5	48.3	57.8	50.4	54.1
7	46.6	34.8	40.2	33.6	34.6	37.0	32.3	34.6
8	73.2	56.6	61.3	43.3	42.9	56.5	47.6	52.1
9	61.5	63.6	68.1	39.3	44.6	60.9	45.5	53.2
10	66.3	55.4	56.4	46.1	44.5	57.1	44.7	50.9
11	35.0	31.8	35.4	34.4	36.3	34.5	33.6	34.1
12	69.5	53.7	66.7	n.a.	60.6	57.5	55.6	56.5
13	58.5	45.2	44.1	43.0	43.7	51.4	42.9	47.2
14	38.7	33.7	38.1	n.a.	35.3	36.9	34.3	35.6
15	36.5	35.1	33.7	33.0	33.1	37.6	34.6	36.1
16	54.1	43.3	60.5	n.a.	50.0	49.9	42.3	46.1
17	46.3	37.8	46.3	37.1	39.6	46.5	40.0	43.2
18	41.3	35.7	36.6	35.0	38.3	40.0	35.9	38.0
Mean	55.6	46.7	49.9	41.1	41.7	47.7	41.0	44.4
SD	13.8	11.7	12.1	7.0	6.9	8.9	6.6	7.6

All values were taken from the right arm. FDP = flexor digitorum profundus; FDS = flexor digitorum superficialis; FPL = flexor pollicis longus; EPB = extensor pollicis brevis; APL = abductor pollicis longus; Ant. = anterior forearm; Post. = posterior forearm; Avg. = average T_2_ from anterior and posterior forearm values. n.a.—Not available.

**Table 5:** Spearman’s rho correlations between clinical tests and FF in the forearm muscles.

	FDP	FPL	FDS	APL	EPB	Ant. FA	Post. FA
HG	−0.82 **	−0.45	−0.61 **	−0.41	−0.17	−0.82 **	−0.60 *
HG pred	−0.82 **	−0.48 *	−0.59 *	−0.36	−0.10	−0.77 **	−0.65 **
LFF	−0.79 **	−0.41	−0.55 *	−0.22	−0.09	−0.74 **	−0.46
UEFI	−0.77 **	−0.68 **	−0.78 **	−0.49	−0.36	−0.81 **	−0.63 *
vHOT	0.45	0.29	0.19	0.28	−0.03	0.18	0.16

HG = handgrip strength; pred = predicted; LFF = long finger flexor; UEFI = Upper Extremity Functional Index; vHOT = video hand opening time; FDP = flexor digitorum profundus; FPL = flexor pollicis longus; FDS = flexor digitorum superficialis; APL = abductor pollicis longus; EPB = extensor pollicus brevis; Ant. FA = anterior forearm; Post. FA = posterior forearm. * Correlation is significant at the 0.05 level (2-tailed). ** Correlation is significant at the 0.01 level (2-tailed).

**Table 6:** Spearman’s rho correlations between clinical tests and T_2_ in the forearm muscles.

	FDP	FPL	FDS	APL	EPB	Ant. FA	Post. FA
HG	−0.75 **	−0.73 **	−0.83 **	−0.72 **	−0.80 **	−0.90 **	−0.90 **
HG pred	−0.81 **	−0.82 **	−0.84 **	−0.74 **	−0.75 **	−0.89 **	−0.92 **
LFF	−0.66 **	−0.66 **	−0.73 **	−0.62 **	−0.79 **	−0.80 **	−0.81 **
UEFI	−0.55 *	−0.69 **	−0.64 **	−0.68 **	−0.67 **	−0.82 **	−0.83 **
vHOT	0.48 *	0.34	0.44	0.27	0.31	0.46	0.37

HG = handgrip strength; pred = predicted; LFF = long finger flexor; UEFI = Upper Extremity Functional Index; vHOT = video hand opening time; FDP = flexor digitorum profundus; FPL = flexor pollicis longus; FDS = flexor digitorum superficialis; APL = abductor pollicis longus; EPB = extensor pollicus brevis; Ant. FA = anterior forearm; Post. FA = posterior forearm. * Correlation is significant at the 0.05 level (2-tailed). ** Correlation is significant at the 0.01 level (2-tailed).

## Data Availability

The data that support the findings of this study are not publicly available due to privacy reasons but are available from the corresponding author upon reasonable request.

## Abbreviations

The following abbreviations are used in this manuscript:

DM1	Myotonic Dystrophy Type 1
ADLs	Activities of Daily Living
qMRI	Quantitative Magnetic Resonance Imaging
FF	Fat Fraction
BMI	Body Mass Index
QMT	Quantitative Muscle Testing
vHOT	Video Hand Opening Time
UEFI	Upper Extremity Function Index
LFF	Long Finger fFexor
HG	Handgrip
FDP	Flexor Digitorum Profundus
FDS	Flexor Digitorum Superficialis
FPL	Flexor Pollicis Longus
EPB	Extensor Pollicis Brevis
APL	Abductor Pollicis Longus
ROI	Region of Interest
